# Problems of Hospital Policy

**Published:** 1911-04-22

**Authors:** 


### PROBLEMS OF HOSPITAL POLICY.

### SIR SAVILE CROSSLEY'S ADDRESS TO THE HOSPITAL SATURDAY FUND.

We have at the present time three large Hospital Funds collecting money for the hospitals of London, and I am very anxious that everybody in the room shall go away with the right impression, which is that the working classes of London contribute a very large proportion toward the amount required. I think that the total amount collected—£42,000—compares very well, if you consider the people who give it, either with the King Edward's Hospital Fund or the Hospital Sunday Fund. I do not say that in the least with any idea of running down the other two Funds with which I have the honour to be connected; but I want everyone to understand, whether rich or poor, that the working classes of London appreciate the benefits they receive from the hospitals, and do their best, in return, to support them and give out of their own pockets toward the benefits they receive. The £42,000 to which I have referred include the amount received in part payment of benefits granted by the Distribution Committee, namely, £6,259, by the Surgical Appliance Committee, £1,276, and by the Ambulance Committee, £88. Thus the total was much larger than that conveyed by anyone glancing casually at the Report, and going away with the idea that the working classes of London only contribute £34,000.

### Local Committees.

With regard to the local committees, they again deserve great commendation. The most noticeable were the five new committees formed—mostly in the West of London—Westminster, Kensington, Hammersmith, Acton and Chiswick, and Penge and Beckenham. We hope that this year a further stimulus may be given to the Fund by the establishment of a very strong Committee for the City of London itself. Up to the present time we have not collected as much money as we ought to have done for that city, and I know very well that there are many firms which would be ready to contribute to the Hospital Saturday Fund if they are only asked. I hope this year a very strong branch of the Fund may be established in the City of London.

### Accounts.

With regard to the accounts, the number of Institutions to which grants have been made is much larger than in the case of King Edward's Hospital Fund. The Saturday Fund goes into many other Institutions, which are for the great benefit of the suffering poor, other than those of the General Hospitals and Dispensaries and Sanatoria which other Funds help. I have especially to thank those who have assisted us to the thirty-seven endowed beds in the Sanatoria.

The demands upon the Fund grow every year, and the work of the office becomes heavier and heavier continually. When I had the honour of becoming the chairman of the Fund ten years ago the working expenses were 11 per cent., but they had been reduced every year since, and had now come down to 7 per cent. If those charitably inclined remembered the enormous amount of work that we have, and the very small sums that are collected in this Saturday Fund—because the pence of the working classes cost nearly as much to collect as the sovereigns from the rich man—they would see they had not much to blame themselves with as regarded the cost of collection.

### The Treatment of Defective Children.

The action taken by the London County Council with respect to school children was a very proper action. It was obviously right that all those defects, which are quite small things in children, should be dealt with before they become serious evils, and before they interfere with the usefulness of those who unfortunately possess them. Now the London County Council has taken the matter in hand, and the Hospital Saturday Fund had good hope of passing a regulation to the effect that subscribers to the Saturday Fund and to similar institutions should not in future be charged part of the cost of the treatment of their children.

### The Out-Patients Problem.

With regard to hospitals and their out-patients, a committee is at present sitting, inaugurated by the King Edward's Hospital Fund, to inquire into the whole of the trouble of the out-patients' system. It would be improper for me, being connected with the King's Fund, to go at length into that question, but everyone who has to do with the hospitals in London knows very well that how far the right people are admitted and how far the wrong ones are admitted is one of the great troubles at the present time. I will only remind you that the hospitals were not meant originally for the use of everyone; the hospitals are meant for those, as a rule, who are not able to pay the whole cost of their treatment. If they are not able to pay anything toward the cost they are Poor Law cases, and if they are able to pay the whole of the cost they would not be fit subjects, and what we want to deal with in the hospitals are those who are able in times of health to pay for themselves, but who are not able to stand the strain of the serious illness of themselves or their families.

On the other hand, there are many cases which must go to the hospital where the local practitioner has failed to, or cannot, cure. These cases you might call the consulting cases, and it is those cases in particular which the hospitals have largely benefitted in the past. I think the Dispensaries of London will be extended very much more widely in the future, and I believe the out-patient question can be dealt with largely by the extension of the present system of Provident Dispensaries.

I am glad to think that His Majesty should be Patron of that Fund, and that we should have the advantage here, not only of His Majesty, but also of the Lord Mayor.

